# Retrospective Evaluation of Invisalign^®^ Mandibular Advancement in Growing Patients: Cephalometric, PAR and 3D Molar Displacement Outcomes

**DOI:** 10.3390/dj13110517

**Published:** 2025-11-05

**Authors:** Teresa Pinho, Carolina Clemente, Inês de Castro, Maria dos Prazeres Gonçalves

**Affiliations:** 1 UNIPRO—Oral Pathology and Rehabilitation Research Unit, University Institute of Health Science (IUCS), CESPU, 4585-116 Gandra, Portugal; a31677@alunos.cespu.pt (C.C.); a28233@alunos.cespu.pt (I.d.C.); 2 Unit for Multidisciplinary Research in Biomedicine (UMIB), School of Medicine and Biomedical Sciences (ICBAS), University of Porto, Rua Jorge Viterbo Ferreira 228, 4050-313 Porto, Portugal; 3 Associate Laboratory I4HB, Institute for Health and Bioeconomy, University Institute of Health Sciences (IUCS—CESPU), 4585-116 Gandra, Portugal; mprazeres.goncalves@iucs.cespu.pt; 4 UCIBIO—Applied Molecular Biosciences Unit, Translational Toxicology Research Laboratory, University Institute of Health Sciences (1H-TOXRUN, IUCS—CESPU), 4585-116 Gandra, Portugal

**Keywords:** orthodontic appliances, removable, mandibular advancement, malocclusion, angle class II, class II, Invisalign, PAR index, mesialization

## Abstract

**Background**: Class II malocclusion is one of the most prevalent dentoskeletal disorders, usually caused by mandibular retrusion. The Invisalign^®^ Mandibular Advancement System is an aesthetic, comfortable alternative to conventional braces for growing patients allowing mandibular projection and dental alignment. This retrospective study assessed the effectiveness of the Invisalign^®^ Mandibular Advancement (MA) System in growing patients with mandibular retrognathia. **Methods**: At treatment onset, seventeen patients were divided into the following two groups according to the aligner system used and cervical vertebral maturation stage: the Invisalign^®^ First group (CVM2), and the Invisalign^®^ Teen group (CVM3, some transitioning to CVM4), all treated with the Comprehensive Package. Treatment efficacy was evaluated through cephalometric analysis, occlusal classification, and three-dimensional tooth movement assessment. Cephalometric evaluations were performed pre-(T0) and post-treatment (TF). In addition, a clinical follow-up during the retention period was conducted to assess post-treatment stability. **Results**: Significant dentoalveolar and skeletal changes were observed in both groups. The Teen group showed greater mesial movement of the lower molars (3.57 ± 1.26 mm) compared to the First group (1.34 ± 0.48 mm; *p* < 0.001). Conversely, the First group showed greater distal movement of the upper molars (2.24 ± 0.64 mm) compared to the Teen group (1.35 ± 0.69 mm; *p* = 0.002). The PAR index showed significant reductions in both groups (*p* < 0.001), and although the Teen group achieved greater improvements, the First group demonstrated a clinically relevant reduction (23.60 vs. 19.43 points), despite two severe cases that did not achieve full Class II correction. **Conclusions**: Both the Invisalign^®^ First and Comprehensive Package for teens systems improved occlusion and skeletal patterns. These findings support the MA System as an effective option for correcting Class II malocclusion during growth.

## 1. Introduction

Class II malocclusion is considered one of the most prevalent dentoskeletal dysmorphoses [1]. Its global prevalence has been estimated at 19.6%, with marked variation across populations. Prevalence is lowest among Africans (6.8%), highest among Caucasians (22.9%), and intermediate among Asians (14.1%) [2]. It often arises from maxillary protrusion, mandibular retrusion or a combination of both [3,4]. The most common cause is mandibular retrusion, present in about 80% of cases. Therefore, its treatment is of special importance because these patients may present several complex clinical conditions [5,6].

Discussions about the effectiveness of early treatment in these patients and its long-term effects are still subjects of debate. However, it is of the utmost importance to address the treatment of Class II malocclusion, considering that, over time, malocclusion tends to worsen and not self-correct [7]. Therefore, determining the optimal timing to start treatment, along with an accurate diagnosis [8], is essential.

The transition phase between mixed and permanent dentition coincides with an intense period of growth, leading to orthodontic and orthopedic changes [9,10,11]. Several studies state that the ideal time to start treatment is at the peak of pubertal skeletal growth (cervical vertebral maturation stages III–IV) [11]. However, various indicators should also be considered, including chronological age, dental development stages, secondary sexual characteristics, and patient-specific growth patterns [8,10,12].

Functional appliances like the Herbst and Twin-Block (TB) [13] promote mandibular growth and a more anterior repositioning to correct dentoskeletal disharmony [4], which improves the cranio-cervical posture of Class II malocclusion in patients who are still growing [14,15]. However, these functional appliances often have bulky structures, poor aesthetics and can also interfere with speech articulation, resulting in non-compliance with treatment [6,16], since the concern for aesthetic treatments on the part of orthodontic patients has increased substantially [17].

Align Technology™ launched the Invisalign^®^ Mandibular Advancement (MA) in 2017, which has become an alternative to previously known conventional appliances [4,18,19]. The primary goals of this appliance, which is intended for growing patients with Class II malocclusion, are to advance the mandible while also realigning the teeth [14].

This advancement of the mandible is performed by the “Precision Wings” which are incorporated into the buccal surface of the removable appliance, between the premolars and the molars in both dental arches [4,20]. Compared to conventional appliances, Invisalign^®^ MA has numerous benefits, including improved aesthetics, comfort and simultaneous performance of orthodontic and orthopedic treatment [18].

These benefits are crucial to promote patient compliance, which plays an essential role in maximizing treatment effectiveness and minimizing treatment duration [7,8].

Previous research on the Invisalign^®^ Mandibular Advancement System has mainly concentrated on specific treatment outcomes, such as cephalometric changes or clinical improvements, often evaluated in isolation. The present study is distinctive in that it combines cephalometric analysis, occlusal evaluation using the Peer Assessment Rating (PAR) index, and three-dimensional digital model analysis. This comprehensive and multifactorial approach provides a broader perspective on treatment effects, offering additional insights into the effectiveness of the Invisalign^®^ MA system in growing patients.

The main aim of this study was to observe the effectiveness of the Invisalign^®^ MA System in growing patients, comparing the effects between Invisalign^®^ First and Invisalign^®^ Comprehensive Package for teens, using traditional cephalometric analysis, occlusal anomaly classification with the Peer Assessment Rating (PAR) index, and three-dimensional evaluation of tooth movement through STL digital models.

## 2. Materials and Methods

### 2.1. Study Design

This study intended to retrospectively assess the effectiveness of the Invisalign^®^ Mandibular Advancement (MA) System in growing patients. Dental and skeletal changes were evaluated through cephalometric analysis, occlusal classification, and 3D superimposition of digital models. Therefore, these analyses were performed at two different timepoints: one before the start of treatment (T0) and another at the end of the treatment (TF). This was a quantitative, retrospective, observational, longitudinal cohort study.

### 2.2. Samples and Eligibility Criteria

Considering the inclusion and exclusion criteria, this retrospective study involved 17 patients, classified into 2 groups according to their stage of growth, child and teen (Invisalign^®^ First and Invisalign^®^ Comprehensive Package for teens, Align Technology, San Jose, CA, USA, respectively). In total, 10 were treated with Invisalign^®^ Comprehensive Package for teens and 7 with Invisalign^®^ First. In both groups, initial and final cephalometric analyses of skeletal changes (FMA, ANB, SNA, SNB and Wits appraisal) and dentoalveolar changes (overjet, overbite, UI/NA, IMPA and interincisal angle) were performed. The demographic and clinical characteristics of both groups are presented in Table 1. The individuals treated with Invisalign^®^ Comprehensive Package for teens were aged between 9 and 16 years (Mean = 11.9; SD = 2.08), while those treated with Invisalign^®^ First were aged between 7 and 10 years (Mean = 8.3; SD = 0.95). In this study, cases with the Mandibular Advancement (MA) feature were initiated in 2017, when Precision Wings first became available with aligners for teen patients. Cases with Invisalign^®^ First were only initiated from 2021 onwards, as Precision Wings became available with the First system at that time.

They underwent orthodontic treatment with aesthetic aligners by a Specialist in Orthodontics and Invisalign^®^ Diamond Provider (T.P.).

Inclusion criteria:

Participants were eligible for inclusion in this study only if they met the following conditions:

- Growing patients with:
  ○ permanent dentition (Invisalign^®^ Comprehensive Package for teens), or
  ○ stable early mixed dentition (Invisalign^®^ First), presenting:
    ▪ mandibular retrognathia (defined using cephalometric criteria, specifically the SNB angle. Values below the normative mean of approximately 80° (±2°), as described by Steiner [21], indicate a posterior mandibular position in relation to the cranial base).
    ▪ at least 1 total molar Class II relation.
    ▪ overjet > 5 mm.
- Patients with completed orthodontic treatment with the Invisalign^®^ MA System.
- Individuals with available and complete cephalometric analysis.
- Patients with STL files before, at the end of the initial series of MA aligners, and at the end of the treatment.

Additionally, cervical vertebral maturation (CVM) stages were used to assess skeletal maturity. In the Invisalign^®^ First group, all patients were categorized as being in CVM2, which corresponds to a transitional phase of growth. In the Invisalign^®^ Comprehensive Package for teens group, most patients were classified as CVM3, indicating a more advanced stage of skeletal maturation. However, three patients in this group were in transition between CVM3 and CVM4. Given that CVM4 was not fully evident in these cases, they were categorized as CVM3 for consistency in the analysis.

Exclusion criteria:

- Adult patients.
- Patients with cognitive or neurological alterations.
- Individuals undergoing treatment with anti-inflammatories, analgesics, or psychiatric medication.

Furthermore, all patients in the study provided informed consent upon entering the clinic, agreeing that their photographic and radiographic records, gathered for diagnostic and clinical monitoring, could be used in research work, articles, and scientific presentations. Their confidentiality was always guaranteed.

### 2.3. Ethical Principles

This research is part of a dissertation project that, as it involved data from patients from a private clinic, required approval from the Ethics Committee of the University Institute of Health Sciences (CESPU), with reference 13/CE-IUCS/2024. Written informed consent was obtained from the parents or legal guardians of all participants. All data (photographic, cephalometric, and STL files) were anonymized and de-identified before analysis, ensuring confidentiality and compliance with ethical standards.

### 2.4. Data Collection Procedures

The duration of orthodontic treatment differed between the two groups, since Invisalign^®^ First patients have a maximum treatment duration of 18 months. The aligners were changed every 7 days and patients were advised to wear them for at least 22 h a day. Therefore, patient compliance is fundamental to maximizing treatment effectiveness.

All the records were obtained using the iTero^®^ Element 5D Plus scanner (Align Technology, Tempe, AZ, USA) and analyzed with the ClinCheck^®^ Pro 6.0 software. The traditional cephalometric analysis was carried out using NemoStudio^®^ Software V.25.0.0.0 (Nemotec, Madrid, Spain). The severity of malocclusions and the treatment’s effectiveness were assessed using the Peer Assessment Rating (PAR) index. STL-format digital models in occlusion were examined using GeoMagic^®^ Control X™ software 2023.1.0 (Oqton, Ghent, Belgium), allowing for the alignment and comparison of dental structures between the initial (T0) and final (TF) timepoints.

All evaluations and measurements were performed in a blinded manner, with the primary evaluator unaware of patient identities throughout the study. The entire assessment process was conducted by a single author and was continuously supervised and reviewed by a second author.

Firstly, the patients’ clinical records (Photographic and Cephalometric) at the start of orthodontic treatment with clear aligners (CA) were analyzed. Through initial (T0) and final (TF) cephalometric analysis, the following variables were collected: FMA, ANB, SNA, SNB, Wits appraisal, overjet, overbite, UI/NA, IMPA and interincisal angle.

The severity of malocclusions and the effectiveness of the treatment were quantified using the Peer Assessment Rating (PAR) index. The PAR index is used to assess the extent of deviation from optimal occlusion and to objectively measure the success of orthodontic treatment by comparing the models before and after treatment. The PAR Index primarily consists of five elements: maxillary and mandibular right, left and anterior segments, left and right buccal occlusion, overjet, overbite and midlines [22]. For this purpose, data was collected and analyzed using Invisalign^®^ Doctor Site. All PAR components were assessed from digital models, except for overjet, which was obtained from cephalometric measurements as described above.

The weighted British PAR index was consistently applied across all cases, ensuring uniform assessment and comparability of results. According to Dyken R.A. et al. [23], the weighted British PAR scores were distributed as follows: the right, left and anterior segments of both the maxilla and mandible, as well as the lateral buccal occlusions (right and left), were given a weight of 1. The overjet score was multiplied by 6, the overbite score by 2, and midline discrepancies were multiplied by 4. The sum of all these components generated the final weighted PAR score. Then the percentage of improvement was calculated using this formula: %PAR = (PAR T0 − PAR TF)/PAR T0 × 100% [24].

The percentage of improvement in the PAR score was then used to categorize the sample into worse or no different (<30% improvement), improved (30% to 70% improvement), and greatly improved (≥70% improvement) [22]. All PAR scoring was performed in a blinded manner, with the evaluator unaware of whether the models corresponded to baseline (T0) or final (TF).

**Figure 1:**
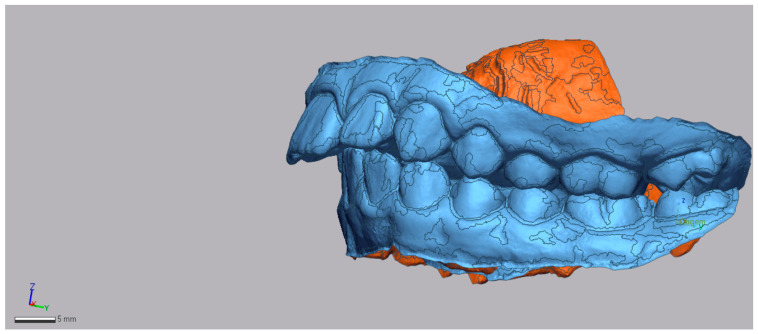
In blue: Example of pretreatment STL with the 3D Mesh, left-side view.

To determine the mesialization values achieved for the first molar, GeoMagic^®^ Control X™ was used using STL files from the beginning and end of treatment. The pre-treatment model of the patient locked in occlusion was imported into the program and a 3D mesh was created (Figure 1).

**Figure 2:**
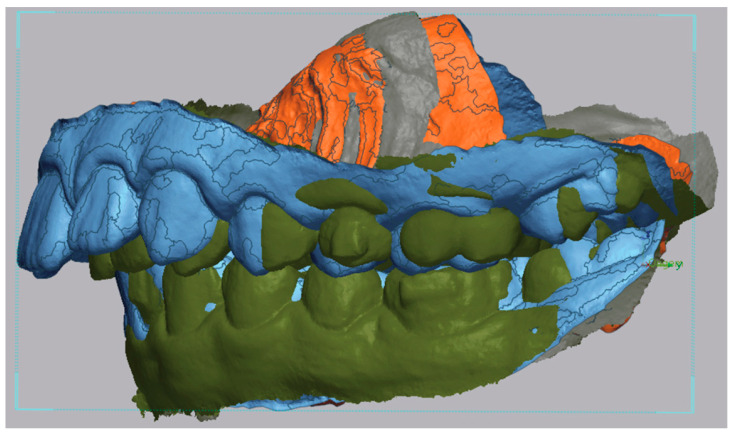
Example of the superimposition of pre- (in blue) and post-treatment (in green) STL, left-side view.

Then the post-treatment STL model was imported and superimposed using the “Initial Alignment” and “Best Fit Alignment” tools (Figure 2). Registration was tooth-borne. The “Best Fit Alignment” used the iterative closest point (ICP) algorithm, applied to palatal rugae and stable dental surfaces (fully erupted first molars and central incisors) as reference areas. Default software settings were maintained, with a tolerance threshold of approximately 0.001 mm, ensuring reproducible alignment across models.

**Figure 3:**
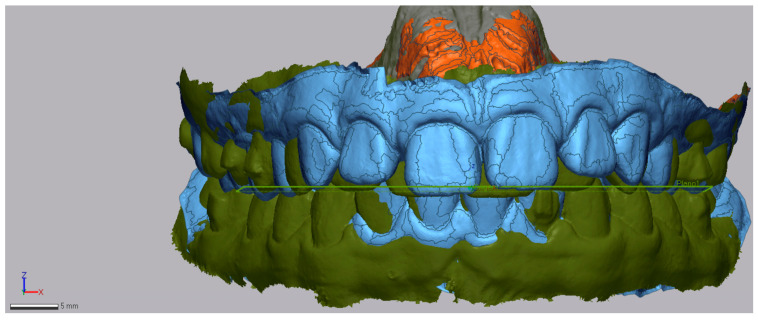
Occlusal plane indicated by the green line in the superimposition of both models.

An occlusal plane was determined using the anatomical references of the mesiobuccal cusp of the upper first molars and the most mesial point on the edge of the upper-right central incisor. This plane was maintained consistently in both the pre-treatment and post-treatment STL models. The initial model helped to define these planes, which then acted as a reference for the final model (Figure 3).

**Figure 4:**
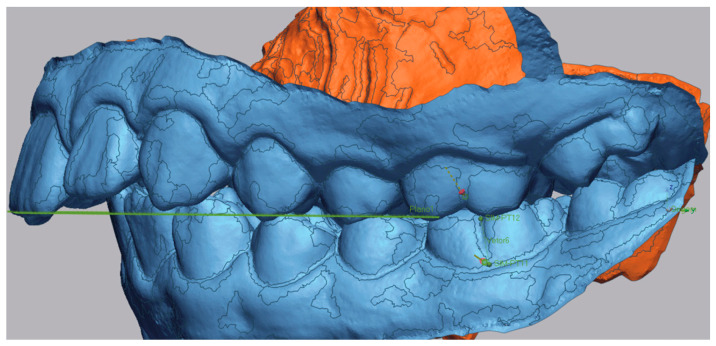
Long axis of first molar of pre-treatment (green line between the two points).

**Figure 5:**
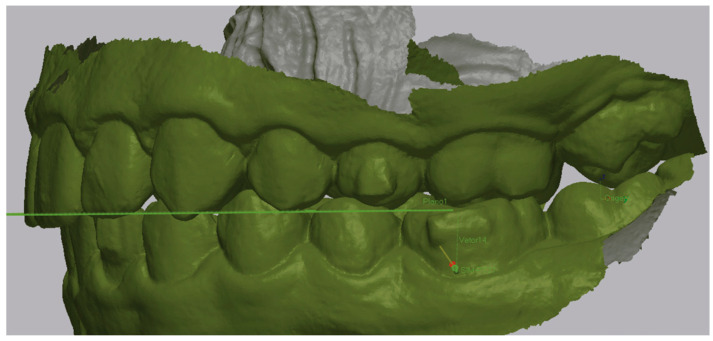
Long axis of first molar of post-treatment (green line between the two points).

Subsequently, the long axis of the molar was traced in both models. A reference point was placed in the sulcus of the first molar, and another was positioned at the most gingival point of the tooth. Vectors were then drawn between these two points to define the long axis of the molar (Figure 4 and Figure 5).

**Figure 6:**
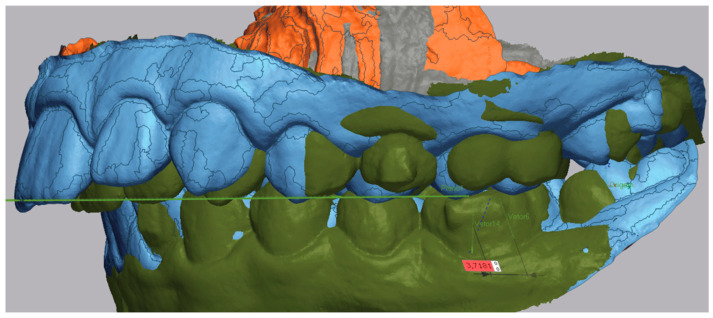
First molar measurements of the mesialization, represented by the black line showing the linear distance.

The distance between both molars axis was measured. Using the ‘Linear Dimension’ tool, the distance between the two vectors of the pre- and post-treatment models is measured (Figure 6).

This measurement reflects the anteroposterior (AP) displacement of the molar axis. The analysis primarily focused on the AP direction, consistent with the clinical goal of assessing molar mesialization. Additionally, the vertical dimension was considered through the evaluation of facial biotype (hypodivergent, normodivergent, or hyperdivergent) at the beginning and end of treatment. However, it should be noted that the transverse dimension was not measured and could not be fully excluded from the analysis, which is acknowledged as a limitation of the study. At baseline, no crossbites or transverse asymmetries were observed clinically.

In addition to skeletal and dental changes, post-treatment stability was assessed. Relapse was defined as a post-treatment increase in overjet of ≥2 mm, or the reappearance of a Class II molar relationship after initial correction. It should be noted that relapse was assessed clinically, not through cephalometric analysis, during follow-up visits, with all patients monitored for at least 12 months (Table 2). During the retention period, occlusal stability, including molar relationship, overjet, and overbite, was also assessed clinically to detect any relapse, without additional radiographic evaluations. After active treatment with aligners, patient adherence to retainer wear was documented based on self-reports and clinical observations during subsequent appointments.

### 2.5. Statistical Analysis

Data analysis was performed using IBM^®^ SPSS^®^ (Statistical Program for Social Sciences) software, version 29.0 for Windows (IBM Corp., Armonk, NY, USA). Descriptive statistics were produced, providing estimates of frequencies and percentages, means, medians, standard deviations, minimums and maximums. The Shapiro–Wilk test was used to assess sample normality, with no evidence of rejection of the null hypotheses. The normality of the data led us to adopt the dependent *t*-test to compare the skeletal changes (FMA, ANB, SNA, SNB, Wits appraisal) and dentoalveolar changes (overjet, overbite, UI/NA, IMPA and interincisal angle) of the initial and final cephalometric analyses with Invisalign^®^ First and Invisalign^®^ Comprehensive Package for teens, as well as the changes in the initial and final PAR index.

To compare the cephalometric measurements, the PAR index, the percentage of reduction, the mesialization (mandible) and distalization (maxilla) between the Invisalign^®^ First and Invisalign^®^ Comprehensive Package for teens systems, the independent *t*-test was used. To measure the magnitude of the effect, Cohen’s d proved to be the most appropriate measure, and, in this sense, the following guidelines were respected: |d| ≤ 0.20 seen as a small effect, |d| = 0.50 as a moderate effect and |d| ≥ 0.80 as a large effect [25].

Although multiple paired and independent *t*-tests were performed, no adjustment was made for multiple comparisons. This decision was based on the fact that each hypothesis tested corresponded to a distinct, clinically relevant outcome defined a priori, rather than repetitive testing of the same outcome dimension. In line with recommendations from the statistical literature, unadjusted *p*-values are reported to avoid an undue increase in Type II error risk, which could obscure potentially meaningful findings in this exploratory context. All *p*-values should be interpreted in the context of the study design and alongside corresponding effect sizes (Cohen’s d), which are provided for each comparison.

The significance level was set at 0.05.

## 3. Results

### 3.1. Descriptive Statistics for Treatment Duration, Number of Aligners and Refinements

The duration of treatment for the Teen group ranged from 20 to 36 months (29.70 ± 6.57), and retention period ranged from 12 to 60 months (24 ± 14.9). The number of aligners used in the MA phase ranged from 40 to 67 (54.40 ± 8.39) with a pre-MA leveling phase included before the Precision Wings, consisting of approximately 24 aligners. The number of additional aligners refinements required to achieve the final result ranged from 1 to 3 (2.0 ± 0.82). Based on the criteria defined for relapse, only 1 of the 10 Teen patients met the conditions for classification as a relapse case. This hyperdivergent patient showed a partial return toward a Class II molar relationship and approximately 2 mm of overjet relapse, which was identified within the first year of retention (Table 2).

After the removal of all attachments, an additional set of aligners without attachments was used, along with Class II elastics for nighttime wear over a period of 1 year, before the retention appliances were provided. In addition, in Teen patients, Class II elastics were used only to facilitate the engagement of the Precision Wings when necessary.

In the First group, the duration of treatment was 18 months for all patients, consistent with the treatment protocol, and the retention period ranged from 12 to 24 months (15.43 ± 5.86). The number of aligners used in the MA phase ranged from 43 to 62 (49.29 ± 6.65) and the number of additional aligners for refinements from 1 to 3 (2.0 ± 0.76).

It should be noted that even before completing the 18 months of aligners, an additional set was requested for stabilization, to be used for at least 1 year with nighttime.

In this group, two severe cases remained in a Class II molar relationship and did not achieve full correction (Table 1). Although the overjet decreased, from 10.2 mm to 6.6 mm in one case and from 8.8 mm to 4.3 mm in the other, both experienced a post-treatment increase in overjet of around 2 mm, which technically qualifies as relapse. Both relapse events in this group were also identified within the first year of retention. However, since full correction had not been achieved initially, these changes should be interpreted with caution. These were severe Class II cases with high initial overjet, and despite the residual sagittal discrepancy, case complexity was substantially reduced and functional outcomes were satisfactory. In these two cases to minimize relapse associated with lower-lip interposition, myofunctional appliances such as Myobrace were used for daily exercises of 1 to 2 h, and patients were referred for myofunctional therapy with a speech therapist.

### 3.2. Skeletal Measures with the Invisalign^®^ Mandibular Advancement

**First system:** At baseline, SNB values ranged from 69.8° to 77.8°. Statistically significant differences were observed between T0 and TF in SNB (*p* = 0.010), ANB (*p* < 0.001) and Wits (*p* = 0.006). The SNB angle measurement showed an average increase of 1.05°, while the ANB angle reduced by an average of 1.84° (*p* < 0.001). Likewise, the Wits index demonstrated a significant reduction of 2.52 mm. On the other hand, the FMA and SNA variables did not show statistically significant differences throughout the analyzed period (*p* = 0.071 and *p* = 0.089, respectively), indicating stability in these parameters. Table 3 presents the results of the analysis of cephalometric measurements relating to the skeletal changes in the Invisalign^®^ treatment with Mandibular Advancement with Invisalign^®^ First system. These findings provide an initial overview of the potential skeletal changes, and further research is needed to assess the significance of these changes.

**Comprehensive Package for teens system:** At baseline, SNB values ranged from 70.9° to 77.4°. Statistically significant differences were observed between T0 and TF in the SNA (*p* = 0.002), SNB (*p* = 0.026), ANB (*p* < 0.001) and Wits (*p* = 0.029). An average reduction of 2.32° in the SNA angle, 2.95° in the ANB angle and 2.50 mm in the Wits appraisal was observed, while the SNB angle increased by 1.03°. On the other hand, the variation in the FMA angle (*p* = 0.237) was not statistically significant, indicating stability in this parameter. Table 4 presents the observed skeletal changes before and after Invisalign^®^ treatment with Mandibular Advancement with teens.

### 3.3. Dentoalveolar Measures with the Invisalign^®^ Mandibular Advancement

**First system:** There was a statistically significant reduction in overjet of 3.83 mm (*p* = 0.005). On the other hand, overbite had a slight increase of 0.63 mm (*p* = 0.587), without statistical significance. The IMPA angle showed a slight increase of 1.74° (*p* = 0.350), without reaching statistical significance. Likewise, the reduction of 5.31° (*p* = 0.196) in UI/NA was not statistically significant. The interincisal angle increased by 4.20° (*p* = 0.415), without significant difference. Table 5 shows the observed dentoalveolar changes before and after Invisalign^®^ treatment with Mandibular Advancement with Invisalign^®^ First.

**Comprehensive Package for teens system:** The analysis of the cephalometric measurements of the initial and final dentoalveolar changes with the Invisalign^®^ treatment with Mandibular Advancement with Comprehensive Package for teens revealed a significant reduction in the mean UI/NA value of 6.54° (*p* = 0.005), accompanied by an increase of 8.29° in the interincisal angle (*p* < 0.001). In addition, a significant decrease in overjet was observed (*p* < 0.001). On the other hand, the change in the IMPA angle (*p* = 0.084) and in the overbite (*p* = 0.070) were not statistically significant, suggesting these variables were not changed significantly throughout the treatment (Table 6).

### 3.4. PAR Index Changes

Analysis of changes in the PAR index throughout treatment with the Invisalign^®^ Comprehensive Package for teens and Invisalign^®^ First systems demonstrated a statistically significant reduction in total scores in both groups (*p* < 0.001). In the Invisalign^®^ Comprehensive Package for teens group, the mean initial PAR index was 27.40 ± 4.90, decreasing to 3.80 ± 3.33 at the end of treatment, with a mean difference of 23.60 points. In the Invisalign^®^ First group, the mean initial index was 28.43 ± 6.45, decreasing to 9.00 ± 5.69, resulting in a mean reduction of 19.43 points. Analysis of the PAR components showed that residual scores in the Invisalign^®^ First group were mainly attributable to persistent sagittal discrepancies, particularly overjet and sagittal molar occlusion (buccal occlusion). In contrast, the teen group demonstrated near-complete correction across all components, resulting in minimal residual PAR. These results indicate a significant improvement in occlusion after treatment with both systems (Table 7).

### 3.5. Differences in Treatment Outcomes Between Invisalign^®^ First and Invisalign^®^ Comprehensive Package for Teens

Comparison of the final treatment results between the Invisalign^®^ First and Invisalign^®^ Comprehensive Package for teens systems revealed statistically significant differences in some parameters (Table 8). Before treatment, there were no statistically significant differences between the groups in the PAR index. However, after treatment, the Invisalign^®^ Comprehensive Package for teens group presented a significantly lower PAR index compared to the Invisalign^®^ First group (*p* = 0.031). Furthermore, the percentage reduction in the PAR index was significantly greater in the Invisalign^®^ Comprehensive Package for teens group (85.54 ± 13.21%) compared to the Invisalign^®^ First group (68.18 ± 15.34%, *p* = 0.025). Regarding tooth movements, the Invisalign^®^ Comprehensive Package for teens group showed better mesial movement of the inferior molars (*p* < 0.001), while the Invisalign^®^ First group showed greater distal movement of the corresponding superior molars (*p* = 0.002), with these differences being statistically significant. These results suggest that, although both systems are effective, differences in treatment objectives between Phase I and comprehensive orthodontic patients may influence the outcomes, and thus, the comparison between the two systems should take these factors into account.

### 3.6. Representative Clinical Cases

**Case 1: Invisalign^®^ First (successful outcome):**

**Figure 7:**
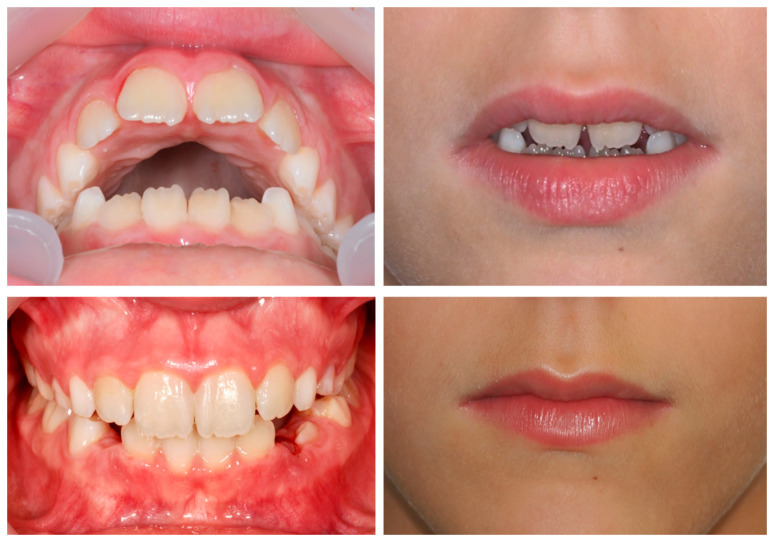
Initial and final intraoral photographs and extraoral lip views of a 9-year-old female patient treated with Invisalign^®^ First.

A 9-year-old female patient presented with a hyperdivergent growth pattern, increased mandibular plane angle, convex profile due to mandibular retrusion, and a slightly closed nasolabial angle. Intraoral examination revealed a bilateral Class II molar and canine relationship, severe overjet and open bite. Treatment with Invisalign^®^ First resulted in marked improvement, with reduction of overjet (from 10.7 mm to 2.3 mm), correction of the sagittal relationship, and establishment of lip competence (Figure 7).

**Figure 8:**
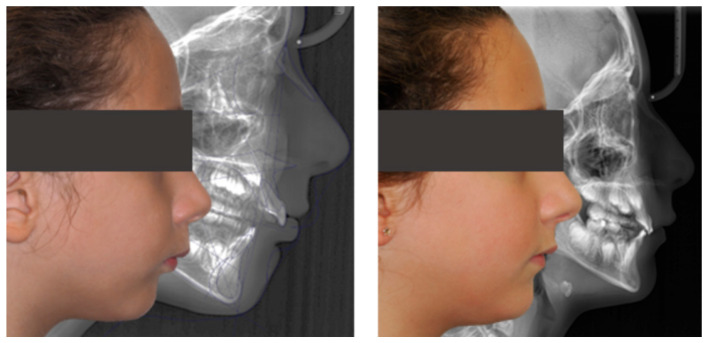
Initial and final lateral photographs and cephalometric radiographs of a 9-year-old female patient treated with Invisalign^®^ First.

Cephalometric evaluation supported these findings, showing a slight decrease in FMA (from 34.9° to 32°), an increase in SNB indicating improved mandibular projection (from 69.8° to 70°), retroclination of the upper incisors (reduced UI/NA values, from 25.4° to 19°), and an increased interincisal angle (from 128.6° to 132.6°), reflecting better dentoalveolar balance (Figure 8).

**Figure 9:**
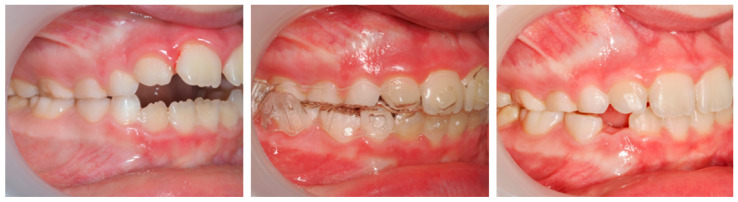
Comparative records at T0 and TF illustrating the clinical progress of the patient (9-year-old female) throughout treatment.

The PAR Index decreased by 94.44%, confirming a substantial occlusal improvement, accompanied by favorable lower molar mesialization (≈2.03 mm) due to mandibular advancement. The clinical progress of this patient is evident in the comparative records between T0 and TF (Figure 9). 

**Case 2: Invisalign^®^ First (partial correction with relapse):**

**Figure 10:**
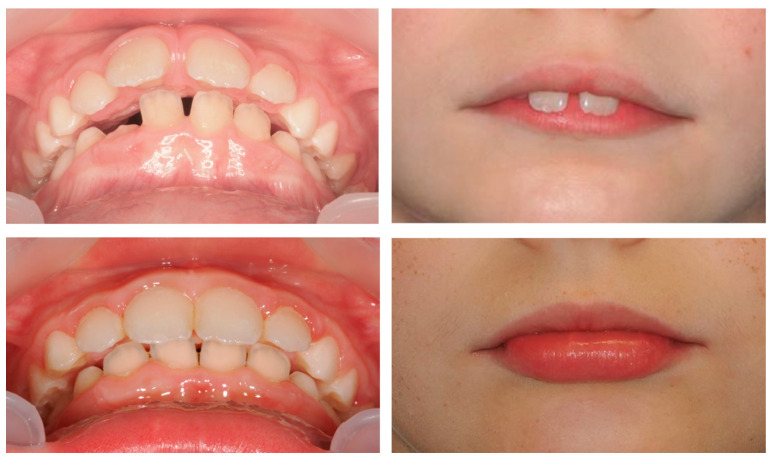
Initial and final intraoral photographs and extraoral lip views of a 9-year-old male patient treated with Invisalign^®^ First.

A 9-year-old male patient presented with a severe Class II Division 1 malocclusion, characterized by a markedly increased overjet (10.2 mm), pronounced upper incisor proclination, lower lip interposition, upper lip hypotonia, and mandibular retrusion. Intraorally, a bilateral Class II molar and canine relationship, and a pronounced curve of Spee were observed (Figure 10).

After 18 months of treatment with Invisalign^®^ First, the patient showed a reduction in overjet to 6.6 mm, flattening of the curve of Spee, and improved lip competence. However, both overjet and overbite remained greater than ideal, the Class II molar relationship persisted, and a mild relapse of approximately 2 mm was observed at follow-up.

**Figure 11:**
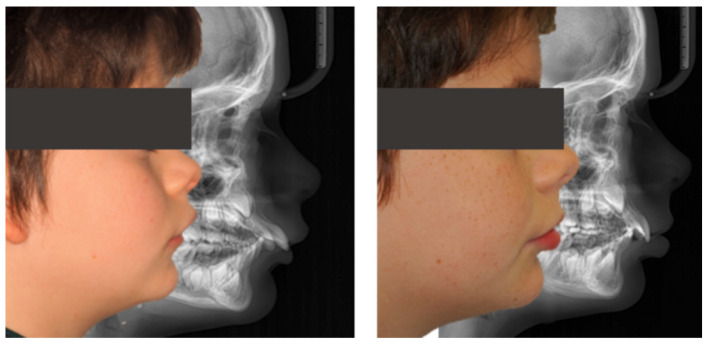
Initial and final lateral photographs and cephalometric radiographs of a 9-year-old male patient treated with Invisalign^®^ First.

Cephalometric analysis showed stability of FMA, a slight decrease in SNA and increase in SNB (from 77.5° to 79.1°), with consequent reduction in ANB. The upper incisors were retroclined (UI/NA from 40.4° to 25.1°) and the interincisal angle increased (from 110.4° to 123.2°), reflecting partial improvement of dentoalveolar balance (Figure 11).

**Figure 12:**
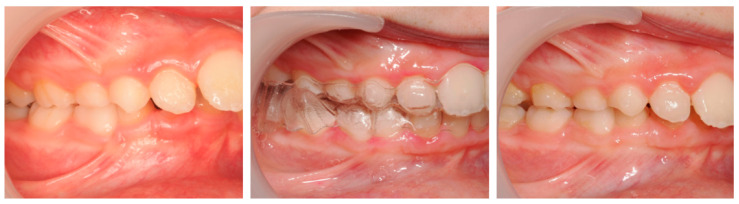
Comparative records at T0 and TF illustrating the clinical progress of the patient (9-year-old male) throughout treatment.

The PAR Index decreased by 44.44%, indicating an improvement in occlusal outcome, although the correction was limited. Mesialization of the lower molars due to mandibular advancement was observed, though less pronounced (≈0.92 mm). The treatment progress is illustrated by the comparative records at T0 and TF (Figure 12).

**Case 3: Invisalign^®^ Comprehensive Package for teens (successful outcome):**

An 11-year-old female patient presented with a skeletal Class II Division 1 malocclusion due to mandibular retrusion, increased overjet, lower lip interposition, and a bilateral Class II molar and canine relationship. Facial analysis revealed a convex profile with marked incisor exposure at rest.

**Figure 13:**
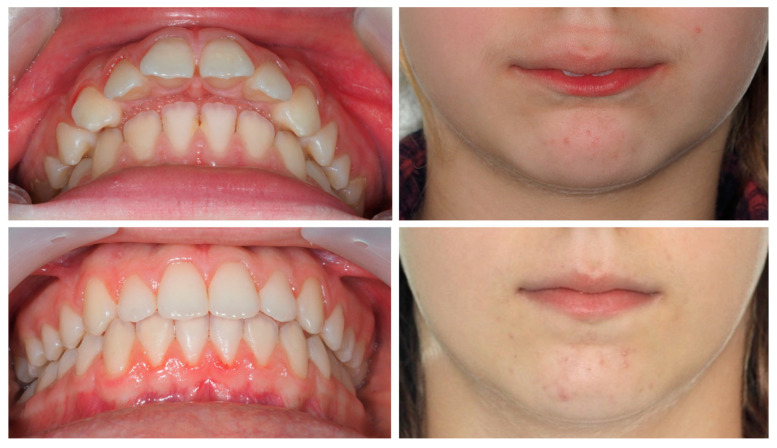
Initial and final intraoral photographs and extraoral lip views of an 11-year-old female patient treated with Invisalign^®^ First.

Treatment with Invisalign^®^ Comprehensive Package for teens using mandibular advancement features resulted in full correction of the sagittal relationship, reduction of overjet to normal values, and improved lip competence and smile esthetics (Figure 13).

**Figure 14:**
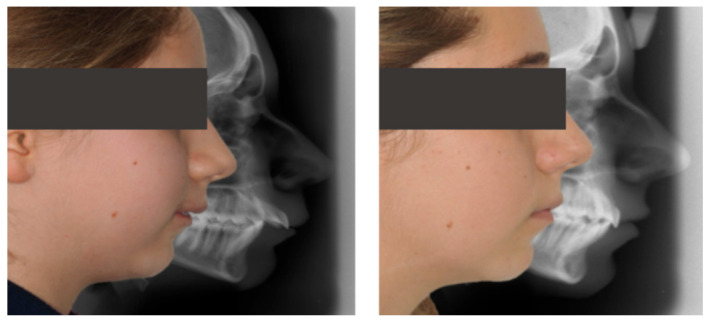
Initial and final lateral photographs and cephalometric radiographs of an 11-year-old female patient treated with Invisalign^®^ Comprehensive Package for teens.

Cephalometric evaluation showed vertical control (FMA decreased from 25.0° to 24.1°), improved mandibular projection (SNB increased from 70.9° to 74° and ANB decreased from 7.2° to 4°), and better dentoalveolar balance (UI/NA decreased from 32.1° to 22.7°, interincisal angle increased from 108.6° to 119.2°), consistent with the clinical improvement in facial profile and lip competence (Figure 14).

**Figure 15:**
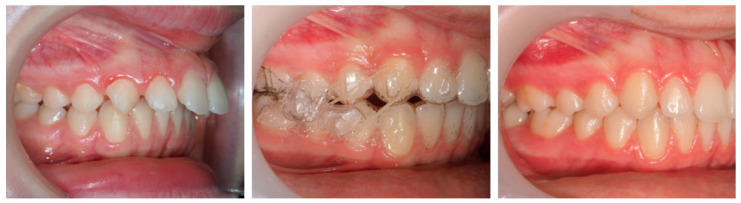
Comparative records at T0 and TF illustrating the clinical progress of the patient (11-year-old female) throughout treatment.

The PAR Index decreased by 93.75%, reflecting a significant occlusal improvement, with good stability and no evidence of relapse, accompanied by satisfactory lower molar mesialization due to mandibular advancement (≈2.87 mm) (Figure 15).

## 4. Discussion

For many years, functional appliances have been used to treat Class II malocclusion, aiming to achieve a skeletal correction of mandibular retrusion, based on placing the mandible in a more anterior position allowing subsequent neuromuscular adaptation [4].

While orthodontic patients aspire to have an aesthetically pleasing smile or profile at the end of treatment, they are unwilling to compromise their aesthetics during the procedure. Consequently, there has been a need to develop more aesthetic, comfortable and easier-to-clean appliances [5,20].

Systematic reviews and meta-analyses of traditional functional appliances, such as Herbst and Twin Block, have consistently demonstrated significant dentoskeletal effects in growing Class II patients, particularly when treatment is timed with the pubertal growth spurt [26,27,28]. This background provides a reference for interpreting the effects of Invisalign^®^ MA.

As a result, the Invisalign^®^ MA System gained popularity, aiming to provide dental, skeletal, and soft-tissue improvements. Its technology features a bite jump-type sagittal mandibular advancement with 2 mm advances every 8 aligners [13].

This treatment begins with a pre-MA phase, which consists of aligning the teeth, and then the MA phase begins. In this phase, the aligners are composed of “Precision Wings”, which will help to promote mandibular advancement [15].

The treatment of Class II malocclusions is therefore extremely important, since failure to carry out interceptive treatment in these growing patients could have severe physiological repercussions for their health and well-being. Timely intervention is essential, considering the peak of pubertal skeletal growth to be the ideal moment, as this is when the rate of condylar development is at its maximum [5]. Evaluating both comprehensive “teen” patients and early intervention “First” patients is necessary to understand how the timing of orthodontic intervention impacts the outcomes. This comparison is important to understand how timing of the Invisalign^®^ MA feature affects the observed skeletal and dentoalveolar changes at two different timepoints.

### 4.1. Dentoskeletal Changes in Patients with Invisalign^®^ First and Invisalign^®^ Comprehensive Package for Teens

For First patients, an average decrease of 1.08° in the FMA angle was observed (*p* = 0.071). For the Invisalign^®^ Comprehensive Package for teens patients, a slight decrease of 0.93° in the FMA angle (*p* = 0.237) was observed. Although these changes were not statistically significant, they suggest potential improvements in the facial biotype, particularly in hyperdivergent patients [15]. This trend is consistent with the Caruso S. et al. study [5].

The reduction in the FMA angle was observed in both groups using the MA system. The presence of plastic material interposed in the arches could contribute to a slight reduction in facial height. In the Comprehensive Package for teens group, this decrease was less pronounced compared to the First group, which may indicate a difference in response to treatment between age groups. However, it is important to note that the observed changes were not statistically significant.

According to the study by Ravera S. et al., a reduction of 2.59 mm in the Wits appraisal (*p* = 0.005) was observed in the CVM2 group, reflecting an early stage of skeletal growth, which corresponds to the stage seen in our Invisalign^®^ First group [7]. Our findings are consistent with these reports, showing a reduction of 2.52 mm (*p* = 0.006). This reduction thus demonstrated an improvement in Class II malocclusion. Likewise, for the Invisalign^®^ Comprehensive Package for teens group, which was classified as CVM3, a significant reduction in the Wits index (2.50 mm, *p* = 0.029) was also observed. This result is in accordance with the findings of Ravera S. et al. [7], who observed a reduction of 3.65° (*p* = 0.008) and aligns with the results obtained by Sabouni W. et al. [8]. While the data suggests a potential effect in correcting the skeletal discrepancy, it is important to note that definitive conclusions regarding the specific impact of Invisalign^®^ MA on skeletal changes require further studies with control groups.

Notably, ANB and Wits are cephalometric indicators for assessing maxillomandibular sagittal discrepancies influenced by dentoalveolar position and growth patterns; however, on their own, they do not prove mandibular lengthening. The ANB angle, originally introduced by Riedel [29], remains one of the most widely used diagnostic parameters in orthodontics. An average reduction of 1.84° was observed in Invisalign^®^ First (*p* < 0.001), supporting a skeletal Class II improvement. In our study, this reduction is in line with the study carried out by Ravera S. et al. in cervical vertebral maturation 2 (CVM2) [7]. In the Invisalign^®^ Comprehensive Package for teens group, a reduction of 2.95° (*p* < 0.001) was observed, which is in agreement with the study carried out by Caruso S. et al. [5] with a reduction of 3.4° (*p* = 0.002), a decrease of 0.91° (*p* = 0.195) that was observed in the study by Ravera S. et al. [7] and a decline of 0.55° (*p* < 0.001) according to Sabouni W. et al. [8].

With respect to the evaluation of the SNB, the Invisalign^®^ First group showed an increase of 1.05° (*p* = 0.010) which agrees with the study carried out by Ravera S. et al. in cervical vertebral maturation 2 (CVM2) [7]. However, these studies, unlike ours, were not statistically significant. The results for Invisalign^®^ Comprehensive Package for teens presented an average increase of 1.03° in the SNB angle (*p* = 0.026), consistent with the studies by Caruso S. et al. [5], Ravera S. et al. on cervical vertebral maturation 3 (CVM3) [7], and Sabouni W. et al. [8]. In the present study, statistically significant differences were observed in SNB, which are consistent with a more anterior mandibular position during treatment. These findings may reflect growth-related or functional adaptations; however, without a control group and without direct mandibular length measurements (e.g., Co–Gn), it is not possible to draw firm conclusions about orthopedic effects. In addition, the results are also supported by the ANB, as discussed previously.

These findings are in line with trends observed in previous studies. Nonetheless, systematic reviews and meta-analyses highlight that the magnitude of mandibular growth stimulation varies according to appliance type, treatment timing, and study design. For example, Cozza et al. and Marsico et al. reported supplementary mandibular elongation mainly when treatment coincided with the adolescent growth spurt, with Herbst and Twin Block emerging as the most efficient appliances [27,28]. Therefore, while our results suggest skeletal contributions, direct equivalence should be interpreted with caution.

In the Invisalign^®^ First system, the SNA did not show a significant change (*p* = 0.089) meaning that the maxilla, in the sagittal direction, remained practically in the same position, which agrees with the study carried out by Ravera S. et al. in cervical vertebral maturation 2 (CVM2) [7]. In the Comprehensive Package for teens system there was a significant reduction of 2.32° (*p* = 0.002). This suggests retraction of the maxilla at the A-point in relation to Nasion, which may be associated with the retroclination of the upper incisors, potentially leading to bone remodeling at the level of the incisor roots and the A-point area [5]. In the study by Sabouni W. et al., the authors also found a decrease in this measurement, although it was not significant as ours [8].

### 4.2. Dentoalveolar Changes in Patients with Invisalign^®^ First and Invisalign^®^ Comprehensive Package for Teens

Given that the initially pronounced proclination of the upper incisors was reduced beyond normative values, resulting in a certain degree of retro-inclination, this correction, although not necessarily ideal, proves beneficial in decreasing incisor exposure, particularly in cases of significantly increased overjet, thereby lowering the risk of incisor trauma. [7]. However, this outcome also highlights one of the most recognized limitations of functional appliances, including the Invisalign^®^ MA System: the challenge of precisely controlling upper incisor inclination [5].

In a limited number of cases (n = 2) within the Invisalign^®^ First group, the Myobrace was used as a supportive appliance in patients who presented residual sagittal discrepancies and lacked functional stability at the end of the 18-month aligner treatment period. Its purpose was to promote functional stability and reduce lower lip interposition in complex cases. In cases where labial incompetence was not yet stable, myofunctional appliances such as Myobrace were used for daily exercises of 1 to 2 h, and patients were referred for myofunctional therapy with a speech therapist. This approach aimed to improve perioral muscle function and long-term stability through interdisciplinary intervention. This complementary intervention did not interfere with the primary treatment outcomes and was part of an individualized retention strategy based on clinical judgment. Its use does not represent a methodological limitation of the study but highlights the value of interdisciplinary care in maintaining partial corrections and preventing further relapse.

Considering the initially normal upper incisor inclination in the Invisalign^®^ First group, there was a decrease in UI/NA from an average of 24.17° ± 10.69° to 18.86° ± 3.13° (*p* = 0.196). In contrast, the Invisalign^®^ Comprehensive Package for teens group exhibited a statistically significant decrease of 6.54° (*p* = 0.005), though the initial upper incisor inclination in this group was above the normal range.

As a consequence of this retroclination, the interincisal angle increased in both groups, aligning with the findings of Ravera S. et al. [7]. However, unlike that study, our results did not reach statistical significance (*p* = 0.415), which may be explained by the behavior of the IMPA angle. Specifically, in the Invisalign^®^ First group, the IMPA angle increased, while in the Invisalign^®^ Comprehensive Package for teens group, it decreased. Although these changes were not statistically significant (*p* = 0.350 and *p* = 0.084, respectively), they align with previous studies [7,8], suggesting that lower incisor inclination was effectively controlled, preventing excessive compensatory proclination.

Regarding overjet, the Invisalign^®^ First group had an initial average of 7.73 mm, which decreased by 3.83 mm (*p* = 0.005), while the Invisalign^®^ Comprehensive Package for teens group showed a reduction of 5.38 mm (*p* < 0.001). Both groups showed significant reductions in overjet. The magnitudes observed fall within or above the ranges reported in individual studies [5,8]. Meta-analyses of functional appliances confirm that clinically relevant overjet reduction is a consistent finding across different treatment modalities, though effect sizes vary with appliance design and timing [26]. In our study, this significant decrease can be attributed not only to the retroclination of the upper incisors but also to the forward positioning of the mandible, as evidenced by an increase in the SNB angle. These findings suggest that the reduction in overjet resulted from a combination of dentoalveolar changes, such as the retroinclination of upper incisors and mesial movement of the molars, and possible skeletal/positional factors associated with mandibular advancement. However, without direct mandibular length measurements and without a control group, an orthopedic effect cannot be directly associated. Moreover, the combination of these dentoalveolar and skeletal factors, including growth, highlights the importance of both dental alignment and skeletal growth patterns in achieving a significant reduction in overjet, rather than dentoalveolar compensation alone.

Another functional parameter that may influence treatment outcomes is the patient’s breathing type (nasal, oral, or mixed). Breathing pattern has been associated with craniofacial growth and occlusal development [30], potentially affecting the response to functional appliances. Since breathing type was not systematically assessed in this study, it was not possible to include it in the analysis. Nevertheless, future research should consider evaluating respiratory function to clarify its role in the effectiveness of the Invisalign^®^ MA system.

### 4.3. Evaluation of the PAR Index Changes

The effectiveness of the Invisalign^®^ Comprehensive Package for teens and Invisalign^®^ First systems was analyzed using the reduction in the PAR index as evaluation scores. According to Richmond et al., for a case to be considered “improved,” a reduction of at least 30% in the initial PAR index is necessary. Furthermore, an average reduction of 22 points in the PAR index is indicative of a “greatly improved” case [31], and a percentage reduction greater than 70% suggests a high standard of orthodontic treatment [22].

Based on these criteria, we analyzed the results obtained. In the Invisalign^®^ Comprehensive Package for teens group, the average initial PAR index was 27.40 ± 4.90, decreasing to 3.80 ± 3.33 at the end of treatment. This represents an absolute reduction of 23.60 points (*p* < 0.001), which exceeds the 22-point threshold predicted for “greatly improved” [31]. In percentage terms, the average reduction was 85.54%, exceeding the 70% limit proposed as a reference for a high orthodontic standard [22]. Thus, the results indicate that treatment with Invisalign^®^ Comprehensive Package for teens not only significantly improved occlusion but did so within the standards of excellence defined in the literature.

In the Invisalign^®^ First group, the initial average PAR index was 28.43 ± 6.45, which decreased to 9.00 ± 5.69, with an absolute reduction of 19.43 points (*p* < 0.001). Although this indicates a significant improvement, it falls below the 22-point threshold suggested for classification as ‘greatly improved.’ The average percentage reduction was 68.18%, approaching the 70% benchmark but not fully reaching it.

This outcome may be influenced by the timing of treatment initiation, as the transition phase between mixed and permanent dentition coincides with an intense period of growth, leading to both orthodontic and orthopedic changes [9,10,11]. Several studies suggest that the ideal time to start treatment is at the peak of pubertal skeletal growth (Cervical Vertebral Maturation Stages III–IV) [11]. Given that the Invisalign^®^ First group includes younger patients who may not yet have reached this peak, their response to treatment could be less pronounced than that observed in the Invisalign^®^ Comprehensive Package for teens group. Additionally, factors such as adherence to aligner use and variability in skeletal maturity should also be considered [8,10,12].

Despite these differences, early intervention with Invisalign^®^ First plays a crucial role in improving function and guiding proper occlusal development. Interceptive treatment at this stage provides significant benefits by addressing malocclusions early, potentially reducing the complexity of future treatments [32]. Although our results indicate that Invisalign^®^ First is less effective than Invisalign^®^ Comprehensive Package for teens in terms of overall PAR index reduction, its contribution to functional improvements and long-term stability should still be considered as valuable.

According to Richmond et al., for a practitioner to demonstrate a high standard of care, the proportion of cases without significant improvement should be minimal, and the percentage reduction in the PAR index should be as high as possible [31]. In this sense, the results for Invisalign^®^ Comprehensive Package for teens indicate a high level of clinical success. Although Invisalign^®^ First demonstrates good results, particularly in functional improvements and stability, in very severe cases that do not achieve full correction, it is hypothesized that extending treatment beyond the current 18-month interceptive phase may offer greater continuity and reinforce long-term outcome stability, a hypothesis supported by two complex cases in our study that did not reach full correction despite marked improvement, highlighting a potential area for further investigation.

### 4.4. Evaluation of L6 Mesialization

The results indicate that L6 mesialization was greater in the Invisalign^®^ Comprehensive Package for teens group (3.57 ± 1.26 mm) compared to the Invisalign^®^ First group (1.34 ± 0.48 mm), with both changes being statistically significant. Additionally, the significant increase in SNB (1.03°, *p* = 0.026) in the Comprehensive Package for teens group reflects the forward displacement of point B, likely facilitating greater lower molar mesialization, leading to a final Class I relation.

Furthermore, the loss of Leeway Space in the transition to permanent dentition could have contributed to the mesialization observed in the lower molars, as this natural physiological drift enhances space closure and facilitates the mesial movement of the molars. This may have played a role in the enhanced mesialization seen in late-transition First cases and in residual transitional effects among Comprehensive Packagefor teens cases, alongside the skeletal changes.

Although distalization of maxillary molars was not a specific treatment objective in the Invisalign^®^ First group, a significantly greater distal displacement of these teeth was observed when compared to the Teen group (2.24 ± 0.64 mm vs. 1.35 ± 0.69 mm, *p* = 0.002). This result may be explained by the stage of mixed dentition in First patients, where the presence of second deciduous molars, which are larger mesiodistally than their permanent successors, preserves the Leeway Space. In these cases, distalization may occur spontaneously or be favored by expansion and decreased anterior pressure as part of overjet correction. This facilitates the recovery of space for retroclination of proclined upper incisors and overall arch coordination.

These findings emphasize the combined influence of skeletal and dentoalveolar changes on treatment outcomes.

In contrast, Invisalign^®^ First is often used in more severe cases of malocclusion, where the primary goal is to be an interceptive treatment, reduce the risk of trauma and improve function, rather than directly focusing on mandibular movement [32]. In this group, two out of seven patients showed relapse due to persistent sagittal discrepancies and a ~2 mm post-treatment overjet increase (one of these relapse cases is illustrated as the second representative case presented in the Results section, Figure 10, Figure 11 and Figure 12). Since full Class I correction had never been achieved, the relapse reflects limited sagittal stability rather than a true regression. These more complex cases, with higher initial PAR scores (28.43 ± 6.45), help explain the increased relapse risk. Still, they showed relevant functional gains and reduced complexity, supporting early interceptive treatment in severe Class II cases, even without full sagittal correction. Importantly, both relapse events in this group occurred within the first year of retention, which is consistent with previous studies indicating that most relapse tends to occur during this period [33], even though follow-up durations varied considerably due to differences in treatment onset.

Additionally, since the patients included in this study were in a growth phase, some skeletal and dentoalveolar changes may have occurred spontaneously, regardless of the treatment. This natural self-correction process is particularly relevant in growing individuals and has been demonstrated in studies such as Tulloch et al., where a small proportion of untreated Class II patients showed favorable skeletal changes. However, the magnitude of these spontaneous changes was generally limited and highly variable [34]. Therefore, while the absence of an untreated control group prevents definitive separation of treatment effects from growth-related changes, the improvements observed in this study are unlikely to be solely explained by natural growth and instead support a treatment-driven effect.

These results are consistent with recent evidence demonstrating the accuracy and clinical reliability of clear aligners in performing complex movements, including distalization and mesialization [35].

### 4.5. Study Limitations

This study has several limitations. The small sample size (17 patients) and its retrospective design may have introduced selection bias, despite predefined clinical criteria. Given the limited sample, the study may be underpowered to detect small effect sizes; therefore, the findings should be regarded as exploratory and interpreted with caution, while still providing preliminary evidence to guide future research with larger cohorts. The young age of participants could also affect compliance, although it was monitored throughout.

Cephalometric analysis is limited by 2D imaging. While 3D tools like GeoMagic^®^ Control X™ improve precision, they depend on digital data quality. In this study, molar mesialization was assessed through linear measurements after model alignment, ensuring internal consistency. Nevertheless, the use of a fixed craniofacial reference point such as Ricketts’ PTV vertical line could provide a more objective evaluation and should be considered in future studies.

The study focused on AP molar displacement and facial biotype but did not assess transverse changes. The lack of a control group restricts comparisons, though ethical concerns prevent exposing untreated patients to radiographs for research purposes alone.

Similarly, post-treatment stability was assessed only through clinical observation, without radiographic follow-up, which limits the precision of long-term skeletal evaluation.

Future studies should include growth-stage matched controls, either from the literature or through synthetic modeling approaches, to better isolate treatment effects from growth.

All evaluations and measurements were performed in a blinded manner by a primary examiner (C.C.). Although no formal calibration indices (e.g., Kappa, ICC) were used, all analyses were continuously supervised and validated by a second author (T.P.), ensuring consistency and agreement throughout the process. However, the absence of formal reliability testing remains a limitation.

### 4.6. Recommendations for Future Studies

Future research should aim to confirm these findings in larger and more diverse samples, which would help improve the generalizability of the results. The application of advanced three-dimensional imaging technologies may provide more detailed insights into skeletal, dentoalveolar and transverse changes associated with the Invisalign^®^ Mandibular Advancement system. Furthermore, prospective longitudinal studies are needed to assess the long-term stability and clinical outcomes of this treatment in growing patients. These directions would contribute to a more comprehensive understanding of the effects of clear aligner–based functional appliances and guide the optimization of clinical protocols.

## 5. Conclusions

The results indicate distinct dentoalveolar and skeletal changes between Invisalign^®^ First and Invisalign^®^ Comprehensive Package for teens. Invisalign^®^ First primarily showed dentoalveolar changes, including overjet reduction and UI/NA angles. In contrast, Invisalign^®^ Comprehensive Package for teens demonstrated both dentoalveolar and significant skeletal changes, such as reductions in Wits and ANB.

Both systems effectively improved occlusion, with statistically significant reductions in the PAR index. However, Invisalign^®^ Comprehensive Package for teens had a more pronounced impact, consistently meeting high standards of care, while Invisalign^®^ First, though effective, showed a slightly less marked response, with a Class I not being achieved in all cases.

Greater molar mesialization in the Comprehensive Package for teens group, alongside an improved PAR index and SNB, indicates a more effective skeletal and dentoalveolar response. In contrast, Invisalign^®^ First focuses on interceptive treatment for severe malocclusions, prioritizing trauma reduction and functional improvement over mandibular advancement.

## Figures and Tables

**Table 1:** Demographic and clinical characteristics of the 2 groups (descriptive statistics).

	Invisalign^®^ Comprehensive Package for Teens Group (n = 10)	Invisalign^®^ First Group (n = 7)
	n (%)	n (%)
Age (Mean ± SD)	11.9 ± 2.08	8.3 ± 0.95
Age range (years)	9–16	7–10
Gender		
Female	7 (70.0)	4 (57.1)
Male	3 (30.0)	3 (42.9)
Initial facial biotype		
Hypodivergent	3 (30.0)	1 (14.3)
Hyperdivergent	4 (40.0)	4 (57.1)
Normodivergent	3 (30.0)	2 (28.6)
Final facial biotype		
Hypodivergent	2 (20.0)	1 (14.3)
Hyperdivergent	2 (20.0)	4 (57.1)
Normodivergent	6 (60.0)	2 (28.6)
Initial right/left molar class		
Class I/II	1 (10.0)	0 (0.0)
Class II/I	1 (10.0)	0 (0.0)
Class II/II	8 (80.0)	7 (100.0)
Final right/left molar class		
Class I/I	10 (100.0)	4 (57.1)
Class I/II	0 (0.0)	1 (14.3)
Class II/II	0 (0.0)	2 (28.6)
Class III/III	0 (0.0)	
Initial Spee Curve		
Normal	4 (50.0)	3 (42.9)
Deep	5 (50.0)	3 (42.9)
Inverted		1 (14.3)
Final Spee Curve		
Normal	5 (50.0)	5 (71.4)
Deep	5 (50.0)	2 (28.6)

**Table 2:** Descriptive statistics for treatment duration, number of aligners and refinements (descriptive statistics).

	Teen (n = 10)			First (n = 7)		
	Min	Max	Mean ± SD	Min	Max	Mean ± SD
Treatment duration (months)	20	36	29.70 ± 6.57	18	18	18.0 ± 0.0
Number of aligners	40	67	54.40 ± 8.39	43	62	49.29 ± 6.65
Refinements	1	3	2.0 ± 0.82	1	3	2.0 ± 0.76
Retention (months)	12	60	24 ± 14.97	12	24	15.43 ± 5.86
Stability n (%)						
No relapse	9 (90.0)			5 (71.4)		
Relapsed	1 (10.0)			2 (28.6)		

**Table 3:** Cephalometric measurements of initial and final skeletal changes—Invisalign^®^ First (dependent *t*-test; n = 7).

					Cohen’s d	
Measure	T0 (Mean ± SD)	TF (Mean ± SD)	Diff (TF–T0)	*p*	Statistic	IC_95%_
FMA (degree)	28.57 ± 7.27	27.49 ± 6.35	−1.08	0.071	0.83	0.07–1.67
SNA (degree)	80.23 ± 3.83	79.44 ± 4.43	−0.79	0.089	0.49	0.32–1.26
SNB (degree)	74.14 ± 3.16	75.19 ± 3.61	1.05	0.010	0.98	0.35–1.87
ANB (degree)	6.10 ± 1.27	4.26 ± 1.51	−1.84	<0.001	0.99	0.74–1.31
Wits (mm)	1.86 ± 0.95	−0.66 ± 1.94	−2.52	0.006	0.77	0.39–0.94

**Table 4:** Cephalometric measurements of initial and final skeletal changes—Invisalign^®^ Comprehensive Package for teens (dependent *t*-test; n = 10).

					Cohen’s d	
Measure/Units	T0 (Mean ± SD)	TF (Mean ± SD)	Diff (TF–T0)	*p*	Statistic	IC_95%_
FMA (degree)	25.36 ± 7.25	24.43 ± 6.19	−0.93	0.237	0.40	0.26–1.04
SNA (degree)	81.29 ± 2.79	79.17 ± 2.82	−2.32	0.002	1.36	0.48–2.21
SNB (degree)	74.73 ± 1.99	75.60 ± 1.99	1.03	0.026	0.84	0.09–1.55
ANB (degree)	6.55 ± 1.53	3.60 ± 1.30	−2.95	<0.001	0.75	0.58–1.44
Wits (mm)	3.12 ± 2.03	0.62 ± 2.50	−2.50	0.029	0.82	0.08–1.53

**Table 5:** Cephalometric measurements of initial and final dentoalveolar changes—Invisalign^®^ First (dependent *t*-test; n = 7).

					Cohen’s d	
Measure/Units	T0 (Mean ± SD)	TF (Mean ± SD)	Diff (TF–T0)	*p*	Statistic	IC_95%_
IMPA (degree)	93.86 ± 6.82	95.60 ± 4.69	1.74	0.350	0.38	0.40–1.14
UI/NA (degree)	24.17 ± 10.69	18.86 ± 3.13	−5.31	0.196	0.55	0.27–1.33
Interincisal angle (degree)	124.19 ± 14.78	128.39 ± 5.79	4.20	0.415	0.33	0.44–1.08
Overjet (mm)	7.73 ± 2.66	3.90 ± 1.89	−3.83	0.005	1.64	0.44–2.78
Overbite (mm)	3.33 ± 3.10	3.96 ± 1.29	0.63	0.587	0.61	0.53–0.97

**Table 6:** Cephalometric measurements of initial and final dentoalveolar changes—Invisalign^®^ Comprehensive Package for teens (dependent *t*-test; n = 10).

					Cohen’s d	
Measure (Unit)	T0 (Mean ± SD)	TF (Mean ± SD)	Diff (TF–T0)	*p*	Statistic	IC_95%_
IMPA (degree)	99.79± 6.52	97.59 ± 5.11	−2.20	0.084	0.61	0.08–1.28
UI/NA (degree)	27.27 ± 6.82	20.82 ± 4.17	−6.54	0.002	1.38	0.48–2.24
Interincisal angle (degree)	117.39 ± 6.54	125.68 ± 4.79	8.29	<0.001	1.55	0.58–2.45
Overjet (mm)	8.55 ± 2.84	3.17 ± 0.83	−5.38	<0.001	1.88	0.81–2.91
Overbite (mm)	4.53 ± 1.27	3.79 ± 1.25	−0.74	0.070	0.65	0.05–1.32

**Table 7:** PAR index changes with the Invisalign^®^ Mandibular Advancement First and Comprehensive Package for teens system (dependent *t*-test; first: n = 7 and teen: n = 10).

					Cohen’s d	
Invisalign^®^ System	T0 (Mean ± SD)	TF (Mean ± SD)	Diff (T0–TF)	*p*	Statistic	IC_95%_
Teen						
PAR index	27.40 ± 4.90	3.80 ± 3.33	23.60	<0.001	4.44	2.33–6.54
First						
PAR index	28.43 ± 6.45	9.00 ± 5.69	19.43	<0.001	2.73	1.04–4.38

**Table 8:** Differences in Treatment Outcomes Between Invisalign^®^ First and Invisalign^®^ Comprehensive Package for teens (Independent *t* test) (independent *t*-test; first: n = 7 and teen: n = 10).

					Cohen’s d	
		Mean ± SD	Mean Diff.	*p*	Statistic	IC_95%_
PAR Index Before	Teen (n = 10)	27.40 ± 4.90	1.03	0.713	0.85	0.79–1.15
	First (n = 7)	28.43 ± 6.45				
PAR index After	Teen (n = 10)	3.80 ± 3.33	5.2	0.031	1.17	0.11–2.21
	First (n = 7)	9.00 ± 5.69				
Percentage Reduction	Teen (n = 10)	85.54 ± 13.21	17.36	0.025	1.23	0.15–2.27
	First (n = 7)	68.18 ± 15.34				
Mesialization of L6	Teen (n = 10)	3.57 ± 1.26	2.23	<0.001	2.18	0.92–3.33
	First (n = 7)	1.34 ± 0.48				
Distalization of U6	Teen (n = 10)	1.35 ± 0.69	0.89	0.002	1.34	0.18–2.45
	First (n = 7)	2.24 ± 0.64				

## Data Availability

Data supporting the findings of this study are available from the corresponding author upon reasonable request.

## Abbreviations

The following abbreviations are used in this manuscript:

PAR	Peer Assessment Rating
STL	STL file format
MA	Mandibular Advancement
